# Letter Regarding Normal Albuminuria in Patients With Autopsy-Proven Advanced Diabetic Nephropathy

**DOI:** 10.1016/j.ekir.2021.12.039

**Published:** 2022-01-13

**Authors:** Samuel N. Heyman, Itamar Raz, Zaid Abassi

**Affiliations:** 1 Department of Medicine, Hadassah Hebrew University Hospital, Jerusalem, Israel; 2 Herzog Hospital, Jerusalem, Israel; 3 Faculty of Medicine, Hebrew University of Jerusalem, Jerusalem, Israel; 4 Diabetes Unit, Department of Endocrinology and Metabolism, Hadassah Medical Center, Jerusalem, Israel; 5 Department of Physiology and Biophysics, Rappaport Faculty of Medicine, Technion-Israel Institute of Technology, Haifa, Israel; 6 Department of Laboratory Medicine, Rambam Health Care Campus, Haifa, Israel

To the Editor:

In the 2021 December issue of the *KI Report*, Sasaki *et al.*^1^ point out that nearly half of 106 Japanese patients with a stage IIa or higher diabetic glomerulopathy, evident on autopsy, were “normoalbuminuric” (urine albumin-to-creatinine ratio <30 mg/g) up to 3 years before death. While addressing this enigma, they ignored the mitigating impact of tubular handling of filtered albumin on albuminuria. Approximately 3.2 g of albumin and additional 10 g of low-molecular proteins are normally filtered daily and are almost entirely uptaken or degraded along the nephron.^2^ More than 70% of filtered albumin is reabsorbed in the proximal tubules in rats, and the rest is uptaken by distal nephron segments. Proximal tubular reclaim of filtered albumin declines in early experimental diabetes in rats,^3^ whereas distal tubular reuptake is reciprocally enhanced.^3^ Tubular uptake of albumin leads to the formation of endocytic vesicles with lysosomal breakdown to amino acids that recirculate in the bloodstream. Paracellular reclaim of albumin likely takes place as well,^2^ as is albumin degradation by brush-border enzymes with urinary clearance of albumin fragments and amino acids.

Thus, diabetic nephropathy may be concealed if filtered albumin is fully reclaimed and degraded, although overt albuminuria reflects glomerular leak of albumin beyond the tubular reabsorptive capacity. Importantly, proteinuria *per se* induces tubuointerstital damage and may reduce tubular capability of albumin reuptake.^4^ Therefore, we urge Sasaki *et al.*^1^ to further evaluate the presence and extent of nonglomerular parenchymal damage in their autopsy samples, anticipating a plausible association of tubulointerstitial disease and albuminuria. We also propose to explore the possible impact of medications that reduce trans-glomerular pressure on the unexpected absence of albuminuria in the presence of structural glomerulopathy.
